# Institutional Capacities Building of Universities in Post-Conflict Context: A Dynamic Managerial Capabilities Perspective

**DOI:** 10.12688/f1000research.173628.1

**Published:** 2026-02-10

**Authors:** Mohammed Abdulqader Mohammed, Hamdan O. Mansoor, Younis M. Kh. Al-Sabaawe, Narmeen AbdulRazzaq Saleh Al-Obaidy

**Affiliations:** 1 dept. of Business Administration, University of Al-Hamdaniya, Mosul, Mosul, Iraq; 2 dept. of Business Administration, University of Fallujah, Al-Fallujah, Al Anbar Governorate, Iraq; 3 dept. Of Business Administration, University of Kirkuk, Kirkuk, Kirkuk Governorate, Iraq; 4 dept. Of Business Administration, University of Kirkuk, Kirkuk, Kirkuk Governorate, Iraq

**Keywords:** Strategic Management, Dynamic Managerial Capabilities, Institutional Capacity Building, Post-Conflict Higher Education, and HEICAT Assessment.

## Abstract

**Background:**

Drawing on the literature on Strategic Management and the Entrepreneurial University approach, particularly through the lens of the Resource-Based View, this study explores how the Dynamic Managerial Capabilities (DMC) of campus leaders foster institutional capacity building in the context of post-conflict higher education. This study aims to develop a clear and detailed understanding of the current status of the University of Fallujah to support post-ISIS recovery efforts while enhancing overall institutional quality. An additional motivation is to assist university administrators in optimally using limited funds and resources by prioritizing core rebuilding areas that can improve institutional performance.

**Methods:**

This study adopts a qualitative case study approach and employs a Higher Education Institutional Capacity Assessment Tool (HEICAT). This study aims to generate specific and concrete evidence on institutional needs that can inform the development efforts of scholars and stakeholders. It also seeks to build capacity among local administrators by supporting their ability to conduct ongoing institutional self-assessments using internationally recognized good practices.

**Results:**

The findings reveal that institutional performance challenges in the case study stem less from material scarcity and more from underdeveloped human, social, and cognitive capital among the campus leaders.

**Conclusions:**

Strengthening the managerial capabilities of campus leaders is essential for advancing institutional adaptability, improving quality assurance, and aligning with international academic standards in post-conflict higher education environments.

## Introduction

Sustained competitive university responses are required in the current global knowledge economy. According to O’Reilly et al. (2019), in the knowledge economy, universities play a vital and growing role in facilitating regional economic development through their three-phase core mission: Teaching and Academic Research, and knowledge transfer activities. Studies have suggested that IT innovation, and yet the absorption of these new technologies, is boosted by education and its ability to improve the quality of human capital ( Aïssaoui, R. and Geringer, M.J., 2018). However, making any sustained response will usually require academic institutions to have capabilities to continuously orchestrate their teaching and research assets, such that collectively those assets remain aligned to changing global needs.

Campus-Leaders play a vital role in university performance ( Leih, and Teece, D., 2016, and Heaton, et al., 2019). However, little is understood about the role of campus leaders in making their universities more successful as well as the factors that mediate their impact. Furthermore, scholars have emphasized that global competition among academic institutions has shifted attention from the organizational process toward the significant role of campus leaders’ managerial skills as a source of university competitiveness. Nonetheless, the mechanism by which campus leaders gain adaptability to international ranking criteria still needs clear insight ( O’Reilly, et al., 2019, and Heaton, et al., 2019).

According to the global education index, Iraq has been removed from the scope of assessment for the last decade as it lacks the criteria for education quality, which qualifies countries to be encompassed (Cited from the Global Competitiveness Report, issued by the World Economic Forum in Davos 2023, World Economic Forum, 2019; Global Competitiveness Report, 2019). Furthermore, based on the QS and THE reports, Iraqi Universities are out of the international ranking for the world's research-led academic institutions ( Top Universities, n.d., Top 1000 - Universities).

This study argues that Iraqi universities have repeatedly been poorly managed. Accordingly, improved leadership and better management of academic institutions are not just necessary, but also vital to improving academic efficiency and productivity. Accordingly, this study focuses on the managerial capability building of educational leadership in Iraqi universities ( Jarzabkowski & Kaplan, 2015). The focus is on the mechanisms by which we can build the managerial capabilities of Iraqi Campus leaders. Three managerial capabilities “key underpinning attributes” will be explored: (a) campus-leaders’ managerial human capital, which indicates learned skills ( Holcomb et al., 2009 P: 459); (b) campus-leaders’ managerial social capital, which refers to managers’ social relationships ( Hitt et al., 2002 P: 354); and (c) campus-leaders’ managerial cognation, which indicates managerial belief systems ( Adner and Helfat, 2003, Helfat and Peteraf, 2015). Based on the foregoing discussion, this research addresses the following research question: How do campus leaders’ dynamic managerial capabilities contribute to institutional capacity building in post-conflict Iraqi universities using the University of Fallujah (UoF) as an illustrative case? The study employs the Higher Education Institutional Capacity Assessment Tool (HEICAT) to diagnose five key performance domains—mission and strategy, academic operations, workforce development, research, and quality assurance—and to explore how leadership behavior, cognition, and social relationships shape institutional outcomes.

The foregoing argument highlights the crucial role of dynamic managerial capabilities in creating, extending, and configuring the academic resources base of Iraqi universities. Therefore, Iraqi universities suffer when they ignore competition and neglect contemporary university strategic management concepts and practices.

This research paper will not be fruitful for Fallujah University, but the advantage might be more generally about how to lead and manage the university to ensure that it maintains its competitiveness capacity, further enhancing its performance in the long term. This study expects to improve the capacity of Iraqi universities to compete with international institutions.

Markedly, applying such a study, particularly in the post-conflict context, will facilitate the university to gain a deep understanding of its strengths and weaknesses inside the university. It also helps the university have a clear insight regarding threats and opportunities in the external environment. Theoretically, this study contributes to Strategic Management and Higher Education scholarship by integrating Dynamic Managerial Capabilities (henceforth DMC) theory with the institutional realities of post-conflict contexts.

The paper proceeds as follows: Section two will review the literature on entrepreneurial universities, strategic management, and dynamic managerial capabilities; Section three outlines the research design and methodology; Section four presents and discusses the findings; and Section five concludes with theoretical and policy implications for rebuilding higher education in fragile contexts.

## Literature review

### The context of universities within the lens of strategic management

Scientific research has become a recognized academic mission within universities since the late nineteenth and early twentieth centuries. The combination of teaching and research remained the dominant university paradigm until the late 1990s, when this “unquestionable” model was influenced by a new dynamic: the emergence of entrepreneurial universities ( O’Reilly, et al., 2019, Al-Sabaawe et al., 2024).

The entrepreneurial role of universities has gradually become accepted as a third mission, alongside the two traditional missions of teaching and research. Etzkowitz and Leydesdorff (2000) argue that this new dynamic represents the emergence of the “second academic revolution,” essentially referring to the transformational process through which public policies sought to transform universities from ivory towers into institutions that were more engaged in the economy and more subject to public accountability. Although the idea of universities collaborating with industry is not new, the second academic revolution was characterized by the establishment of a formal institutional framework for such collaboration through public policy ( Jacobsson, 2002). Etzkowitz (2013) identifies three stages of entrepreneurial university development.

Stage 1 (University Entrepreneur I): The academic institution begins to adopt a strategic vision for its overall direction and acquires a partial ability to determine its priorities, either by generating its own resources through donations, tuition fees, and grants or by negotiating with funding agencies. Stage 2 (University Entrepreneur II): The university plays an active role in commercializing the intellectual property generated by the activities of faculty, staff, and students, thus strengthening its relationship with the knowledge-based economy ( Siegel et al., 2007). Phase Three (University Entrepreneur III): The university plays a proactive role in improving the effectiveness of the regional innovation ecosystem, often in collaboration with the industry and government. The university seeks to become an economic and developmental actor in its local environment. While these phases typically occur in the aforementioned sequence, they may develop in a different order or simultaneously, with the university directing its intellectual resources toward transforming knowledge into economic outcomes without losing its own intrinsic cognitive value.

The forthcoming idea highlights that entrepreneurial universities represent a fundamental shift in the philosophy of higher education, from a model focused on producing knowledge for the sake of knowledge to seeking to harness knowledge as an economic and social force contributing to sustainable development and regional innovation.

### University campus- leaders and institutional change

According to Leih and Teece (2016), the concept of leadership in the university context is closely intertwined with strategic management, as both influence resource generation and allocation. Cyert R. 1990, p. 29 defined leadership as "the ability to focus the attention of organizational participants on problems deemed important by the leader.” Many researchers have recognized the essential role of leadership in university success. However, other researchers have noted that the control of university leaders is limited by external events and the complex nature of university decision-making, in contrast to the greater influence often wielded by senior leadership teams in for-profit institutions ( Leih, and Teece, 2016 P: 185). Apart from the debate over the true extent of influence, some argue that university administrators have become overly focused on external pressure, financial stability, and ensuring efficiency and accountability. This focus is no longer sufficient to meet modern challenges. Therefore, scholars have suggested the Dynamic Managerial Capabilities Framework as a new approach ( Adner & Helfat, 2003; Teece, 2007; Helfat & Martin, 2015). Consequently, supporting university leadership effectively identifies opportunities, sets priorities, implements strategies wisely, and transforms quickly.

### Dynamic managerial capabilities in the context of higher education

Dynamic managerial capabilities (DMC) are derived from general dynamic capabilities theory and refer to the “abilities managers possess to build, integrate, and reconfigure organizational resources’ ( Adner & Helfat, 2003; Helfat & Martin, 2015; Teece et al., 1997). These capabilities enable managers to modify firms' resource bases and determine how to adapt, renew, and maintain their competitive advantage in dynamic and changing environments. Therefore, the variance in performance outcomes across firms is strongly linked to differences in managerial decisions and the ability to reconfigure resources. Superior dynamic managerial capabilities enable managers to reshape their organizations more effectively in line with changing environmental conditions and direct internal resources toward external opportunities.

The literature highlights three key factors that constitute the core pillars of managerial dynamic capabilities: human capital, social capital, and cognition ( Adner & Helfat, 2003). Each of these elements contributes in a unique way to enabling managers to sense, seize, and transform opportunities within their organizations.

Managerial human capital includes knowledge, skills, and expertise acquired through education, training, and prior work experience, which forms the basis for effective decision-making and resource management. Differences in human capital among managers generate variance in performance outcomes at the firm level and can represent a source of competitive advantage if they are rare, valuable, and difficult to imitate or replace ( Castanias & Helfat, 2001; Helfat & Peteraf, 2015).

Managerial social capital refers to the network of relationships and connections that enables managers to access valuable information, resources, and opportunities. Through internal and external connections, managers can enhance information flow, improve collaboration, strengthen the organization's decision-making capabilities, and improve its performance ( Hitt et al., 2002; Adner & Helfat, 2003).

Managerial cognition represents mental models, belief systems, and cognitive structures that shape how managers perceive, interpret, and respond to strategic challenges. Cognitive capabilities influence decision quality, strategic insight, and the ability to adapt to environmental changes ( Adner & Helfat, 2003; Helfat & Peteraf, 2015).

### Institutional capacity building in post-conflict contexts

Capacity building in higher education is defined as “a process aimed at enhancing the capabilities of individuals, institutions, and systems within the higher education sector to perform their functions, solve problems, and achieve goals in a sustainable manner” ( Power et al., 2015, pp. 45–46). This process includes activities that contribute to raising the quality of education, scientific research, management, and governance within higher education institutions, thus enhancing their role in achieving national development goals, particularly in low- and middle-income countries ( Van Deuren, 2013).

According to Power et al. (2015), capacity building in higher education comprises three main components: human capacity, which includes the skills, competencies, values, and attitudes of academics, administrators, and researchers. Institutional capacity: This includes administrative and governance systems, infrastructure, and regulatory and policy frameworks. Environmental Capacities: These include the societal, physical, and contextual factors that create a supportive environment for higher education institutions ( Weber & Duderstadt, 2004).

## Research methodology

### Research design

Based on Yin (2003, 2009, 2018), a single-case design is adopted to examine how the dynamic managerial capabilities of campus leaders enable institutional capacity building in a post-conflict environment. According to Eisenhardt (1989), case study design allows a deep contextual understanding of leadership behavior, resource orchestration, and capability development within a fragile institutional setting.

### Research context

The University of Fallujah, located in the city of Fallujah in the Anbar Governate of western Iraq, supports tertiary education in Fallujah and the surrounding areas in this city. The university was in infancy in 2014, as Da’esh first swept through this city, plunging the region into years of occupation and conflict. Following the defeat of Da’esh, the University of Fallujah leaders aim to rebuild the physical and human capacity of the university.

### Research methods

Drawing on the literature on Strategic Management and the Entrepreneurial University approach, qualitative and quantitative tools were used for collecting and analyzing data. The Higher Education Institutions Capacity Assessment Tool will be used as the primary tool to collect data ( IREX, 2016). HEICAT is a very flexible analysis tool (self-assessment method) that offers a qualitative data collection framework. Furthermore, it provides a quantitative matrix for scoring university performance, as well as a template for providing a detailed view of strategic analysis. Consequently, the tools will precisely diagnose the strategic position of the academic institutions under consideration across a range of academic functions, and consequently enable the administration to formulate strategic plans to gain and sustain their long-term performance.

This approach is informed by the dynamic-managerial-capabilities perspective, which views leadership as an ongoing act of interpretation and coordination under constraints ( Adner & Helfat, 2003; Teece, 2007; Helfat & Martin, 2015). Ethical clearance was secured from the university and the participants provided informed consent. Combining several data types allows for both validation and reflective engagement by institutional actors.

### Study sample and data collection

This study was based on the application of the Higher Education Institutional Capacity Assessment Tool (HEICAT) at the University of Fallujah from December 2019 to January 2020. The assessment process was carried out using a systematic approach for sample selection and data collection to ensure a realistic representation of the university's academic and administrative leadership levels.

Purposive sampling was used to include key academic and administrative leaders involved in the university's post-conflict reconstruction. The number of participants was (40) individuals, which was distributed as follows:

University President, Vice Presidents (for Academic Affairs and Administrative Affairs), Deans and Vice Deans of Colleges, Heads of Academic Departments, Administrative Staff Members representing Quality Assurance, Scientific Research, Planning, and Career Development Units.

The data collection process was supervised by ten lead facilitators, each of whom was responsible for one of the instruments’ five domains: Mission and Strategic Planning, Academic Operations, Workforce Development, Scientific Research, and Quality Assurance. Data collection was based on the HEICAT questionnaire, consisting of 240 items, supported by semi-structured interviews, document analysis, and guided discussion sessions. The questionnaire covered university governance and administration, academic processes, strategic planning, scientific research management, and quality assurance systems.

Facilitators conducted interviews within their respective fields of expertise. The aim was not to present all items to every participant but rather to tailor questions to the respondents’ experiences while ensuring data saturation through the inclusion of multiple perspectives. These responses were supported by documentary and field observations.

### Data analysis

**Figure 1.**
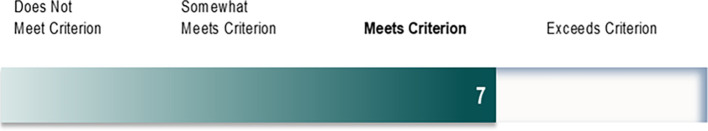
Scoring matrix.

After the data were compiled, the facilitator completed a Scoring Matrix using a Likert scale, which assessed the extent to which the “good practice criterion” was met across a range of indicators in each category. Averaging these scores produces a ‘headline’ performance score for each category. For each category, qualitative feedback was also provided, which highlighted particular areas of weakness, strength, and innovation, and identified key barriers to development. Please see Figure 1.

## Results

**Figure 2.**
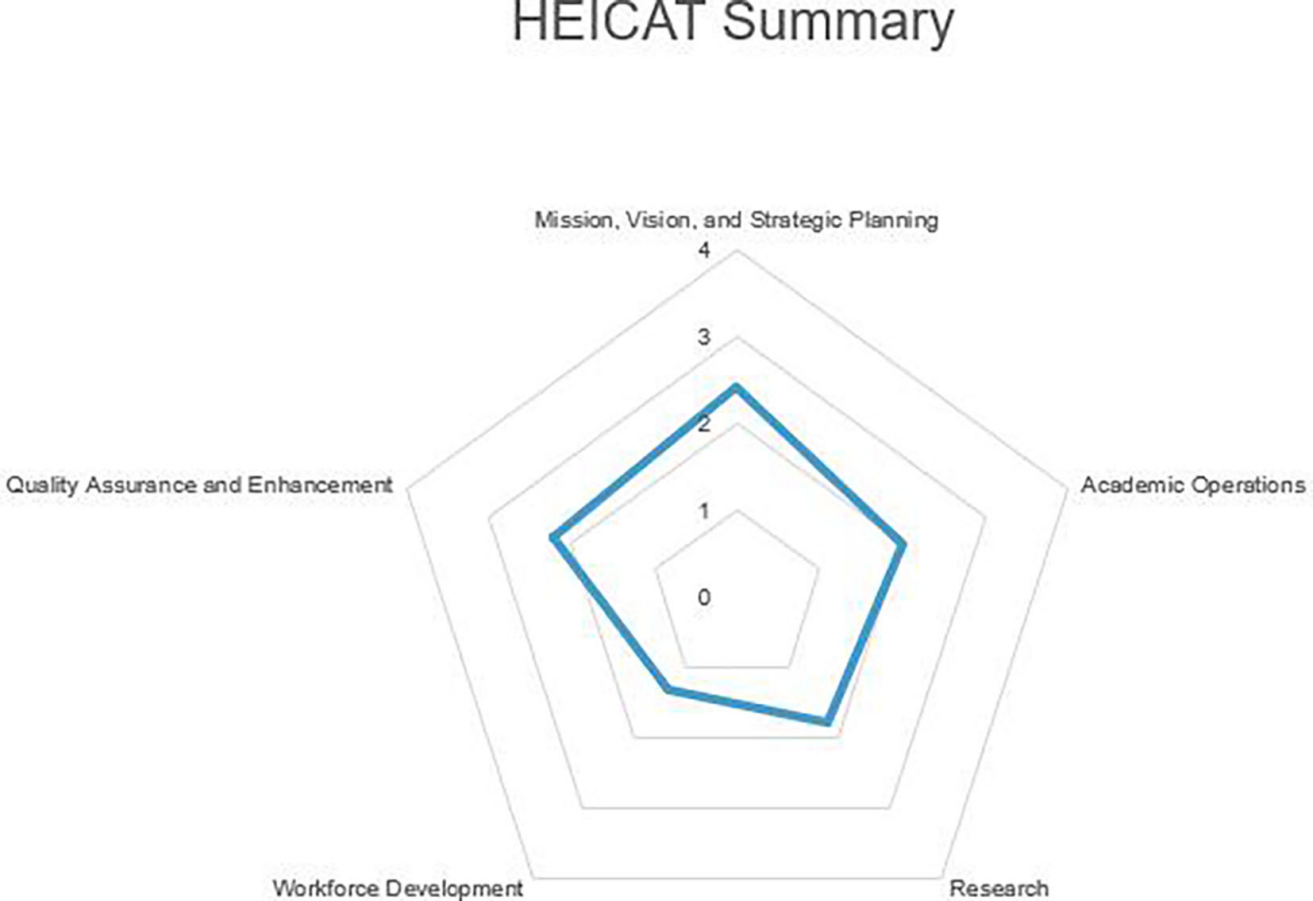
Summarize the main finding of data analysis.

The findings were grouped according to categories in the questionnaire. Please see Figure 2.

### Mission and strategic planning

Development needs

The university has a mission, vision, and strategic plan; however, these are not available for a full review. Based on facilitator feedback, the mission in its current state does not tie in all areas of the university, as many institutional practices do not support the university in meeting its overall institutional goals. The strategic plan is not widely known outside of high-level leadership; in fact, many review committees were unaware of the content of the strategic plan. It was noted that the senior administration is currently working toward an updated institutional plan; however, the draft version was not available for full review. Each college has developed individual missions, visions, and goals; however, they continue to operate independently, and current versions do not tie academic functions or colleges to overall institutional goals. The goals and objectives shared in academic colleges are not clear or measurable; this will need revision to be able to measure progress toward meeting goals. While some areas remain a priority, other stated goals may need to be reviewed for relevance, given the current environment, (see Figure 2).

Strengths

**Figure 3.**
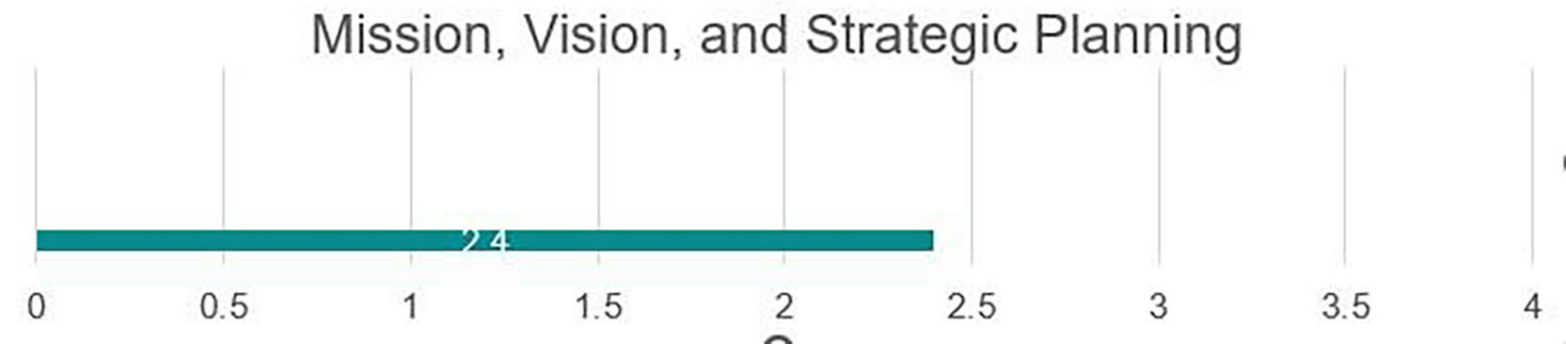
Strategic planning in UoF.

While not necessarily aligned with the institutional mission, each college currently has its mission, vision, and strategic goals. These can serve as a basis for which the university can build, conducting vertical alignment of individual college goals with the institutional mission. Forced rebuilding due to the destruction experienced under Da’esh, while incredibly difficult for everyone within Fallujah, also provides an opportunity to restart the university. The university was in its infancy at the time of Da’esh invasion, and practices, policies, and approaches to higher education in this university are still in the process of being built, allowing the university to avoid challenges or issues that may be experienced in other older universities. Centralized policies and procedures under MOHESR are widely known, followed by faculty members. The university administration is engaged and active with a demonstrated commitment to building a high-quality educational institution. Please see Figure 3.

### Academic operations

Development needs

As we can see from Figure 3, there remains a need to advocate for greater autonomy over various academic operations of the university, including the academic calendar, curriculum, and assessment, as many of these aspects remain centralized. All academic programs would benefit from developing detailed student learning outcomes, along with alternative strategies for the assessment of learning. In today’s global market, academic programs must focus on building frameworks to support the development of student competencies in line with real-world needs. Once frameworks are developed, the alignment of instruction and assessment needs to be mapped to ensure graduates have baseline competencies that need to be successful locally and globally. Programs would benefit from robust faculty professional development initiatives to support more applied hands-on instructional strategies. Currently, few formal communication streams support overall connectedness across academic programs. Additionally, instruction currently relies solely on classes conducted in person on campus with a few alternate modes of instruction, such as online courses.

Strengths

**Figure 4.**
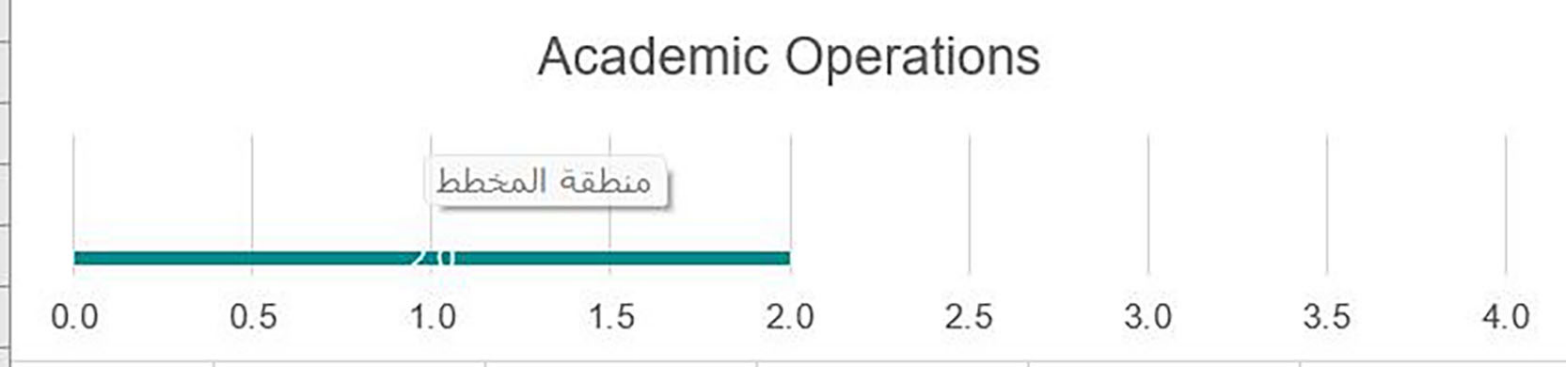
Academic operation In UoF.

Academic Operations were the strongest area for the University of Fallujah, with many systems and processes in place despite the disruption caused by Da’esh, providing some information for prospective students as well as limited opportunities for support services. The university has a set of uniformly applied regulations and procedures for grades, degree pathways, processes for credit, etc., that are known to most in the community. University policy and procedure manuals exist, and information is communicated clearly. There is a commitment by the core team of faculty and administrators to invest in improvements in the curriculum and programs. This commitment is evident in the review team during the review process. There is a need to broaden and institutionalize the approach through full university leadership, (see Figure 4).

### Workforce development

Development needs

**Figure 5.**
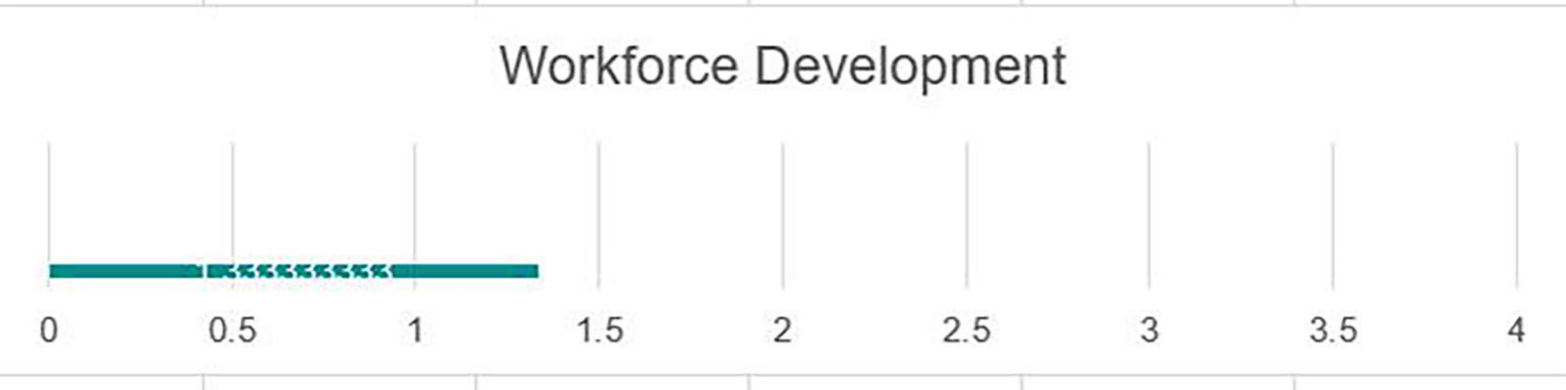
Workforce development in UoF.

The establishment of a Career Development Center, while a key step forward, needs to be augmented by a detailed labor market analysis of the local, national, and global markets to better tailor student support services to real-world needs and opportunities. While there is awareness by the review team of the University Career Development Center, much of the responsibility for work readiness skills acquisition of students seems to fall on the CDC staff. There is a need for academic programs to support applied learning models that lend well to overall workforce development components. The institution could consider embedding ‘transferable skills’ into its curriculum (e.g., through additional courses or specific classroom, teaching, learning, and assessment approaches). While there are few private sector companies in the local Fallujah area, there is a need for closer collaboration with the local market to help shape the development of teaching and learning models that support the close alignment of the relevance of UoF programs to market needs. This collaboration could include the integration of college- or departmental-level advisory panels to provide substantive input for programming. Currently, there are limited avenues through which employers engage in course delivery. The expansion of initiatives such as internships, student innovation projects, design days, guest lectures, site visits, and staff exchanges are needed to further build mutually beneficial ties to the private sector, (see Figure 5).

Strengths

The university has a career development center that trains students in various work readiness skills. Additionally, faculty at various levels are realizing the need to change the cultural mindset at the university level and integrate employment skills into the student experience. The UoF recently participated in a student innovation competition titled ‘Design Day’ to support applied learning. The UoF faculty and CDC staff can build on this experience to expand this real-world experiential learning model to support students’ work readiness, (see Figure 5).

### Research

Development needs

**Figure 6.**
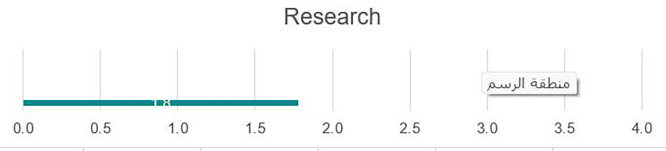
Research at UoF.

Figure 6 illustrates that the university would benefit from the development and dissemination of an institutional research strategy that includes priority areas or fields for research, incentives for faculty research, available resources, and evaluation/promotion criteria based on research outputs. A tiered approach for developing institutional research that encompasses undergraduate research, the development of robust research methodology courses, graduation requirements for student research, and other mechanisms to train future researchers, to build on the previous point, integration of student support positions such as Research Assistants or Graduate Assistants, would provide support for faculty while creating a mentoring environment to support young researchers in their professional growth. To facilitate research growth, a research management office is needed to assist faculty in areas such as locating funding opportunities, managing grants, and other aspects of research management. There is a need to develop a more enabling environment for top researchers that would allow time to focus on institutional research priorities. Examples include the release of time from teaching, small stipends or payments for research, and other incentives. There is a need to develop and institutionalize a research ethics policy to discourage plagiarism by faculty and students and ensure do no harm approaches in research; faculties who may be good researchers are not necessarily effective grant writers and would benefit from institutional support in applying for research grants, fellowships, or other opportunities, which could be incorporated into the curriculum in various disciplines to allow for opportunities for students to conduct basic research during academic study. There is a core need for applied research in various disciplines and fields in post-Da’ esh recovery, particularly in areas such as sociology or psychology. The pre-Da’esh infrastructure to support research had numerous gaps that were exacerbated by the destruction of labs and equipment under Da’esh control.

Strengths

Research and publications are system-wide priorities with an existing criterion for faculty promotion; therefore, it is widely known that faculty should engage in academic research, (see Figure 6).

### Quality assurance and enhancement

Development needs

**Figure 7.**
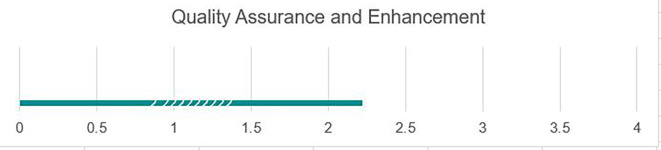
Quality assurance and enhancement at UoF.

Student records and other key data were kept in hard copies, and many were destroyed. Faculty and administrators currently keep data in paper-based hard copy formats, making it difficult to draw from data for assessing overall university quality of services, as the information and files can be so large that they can be difficult to fully review, as shown in Figure 7. Currently, universities do not use data for decision-making. The University of Fallujah administrators would benefit from open-source electronic data visualization software such as Tableau to review high-level data for decision-making and understanding trends over time. While the quality assurance process is in place, there is a need to develop a comprehensive quality enhancement process that draws from quality assurance findings to systematically map out an ongoing quality improvement plan.

### Strengths

As shown in Figure 7, the university has robust systems and processes in place to collect and review data on institutional quality that are widely known across the institution; each college currently has a quality assurance unit in place responsible for data gathering, is currently in the process of revision, and contains a great starting point to access information. Various teams and units across campuses understand the current data collection process and can continue with little training.

## Discussion

Conduct a full strategic planning process to develop goals and objectives that fit the current context in which the university operates. Post-Da’esh circumstances will require the university to be nimbler in adapting to the local environment. One crucial part of the planning process is the incorporation of stakeholder input from the local community, including students, industry, and local government. Identify well-known international universities with similar size, demographics, and focus to serve as benchmark institutions for strategic growth. Choose key areas to gauge progress toward standards identified in the benchmark institution. Conduct a full environmental scan of the post-Da’ esh community, services, institutions, etc. Develop strategies for effective university communication to ensure everyone is aware of the institutional mission and focus, provide updates on the development of the university and surrounding areas, and foster a general feeling of connectedness to the institution. This could be through basic email listservs, with updates from the president’s office or senior administrators being shared weekly, stories of the university being highlighted, etc. Communicate university strategic plans, goals, and objectives broadly across campuses, including clear expectations for faculty and staff in reaching institutional goals. Institutional data collection, storage, and use are important for decision making and strategy development. Develop and operationalize an Institutional Research Office to support administrative decision making by utilizing appropriate data. Conduct comprehensive management training and professional development for academic leaders within the UoF.

Considers the integration of an institutional communication system to share key information, updates, and the overall connectedness of faculty and students. This could include enhancing the university website to repair broken links, providing substantive information on student support opportunities, and sharing key information.

1. Each department will need to develop robust student learning outcomes and multiple assessment practices to ensure that learning takes place in each department that equips graduates with the skills needed to be successful in the current local and global job market.
2. Each department should work with the senior administration to develop guidelines to ensure that they are prioritizing areas that support the university mission, vision, and goals. Colleges should align academic operations to support overarching university missions, visions, and goals as institutions.
3. Develop ongoing professional development training for faculty to support growth. This would ideally be done through an established office for professional development; however, it can be achieved with few resources by having faculty who have received training in a specific field or area conduct seminars and hands-on training for peers.
4. Benchmark key academic departments of a U.S. university academic department of similar size and scope to map out essential areas for growth in line with international quality standards.
5. Align student academic experience with university initiatives, such as the Career Development Centre to support the development of well-rounded, highly skilled graduates. For example, integrating hands-on learning through design-day initiatives, Summer Internships, and work readiness training into curriculum and departmental academic activities.
6. Pilot integration of online instruction or virtual classrooms through existing courses to develop alternate modes of instruction and support acquistion digital skills in students.
7. Consider models for electronic student management systems for academic operations.
8. Develop external advisory panels or committees to ensure that academic programs meet local needs. For example, departmental industry advisory committees can ensure that classroom learning more closely models real-world experiences.

CDC staff can conduct strategic planning exercises to build a systematic approach to broadening the UoF institutional approach to workforce development. For example, creating an annual plan that maps out key areas of engagement, activities, and publication of services to broaden awareness of the center and services on campus. Engage key academic faculty and administration in the upcoming labor market survey process to broaden the institutional understanding of job opportunities in the surrounding area, in addition to fostering industry relationships with academic programs for further, sustained engagement, Work with CDC staff to conduct orientations for academic faculty on CDC services for overall awareness as well as ways in which academic programs and faculty can support skills acquisition in students, and develop an institutional system for tracking graduate employment and maintaining engagement with the university. This could be done through an alumni unit or office that works in coordination with the CDC and academic departments.

Develop a comprehensive research strategy based on the current context of Fallujah, including the launch of a Research Management office, support for researchers in identifying funding resources, creation of research method courses, priority fields for applied research to support community building, faculty incentives for conducting research, and other areas. Create and disseminate a university-wide research ethics policy; incentivize faculty research by reducing teaching load and other requirements that allow faculty to focus on research; create professional development opportunities for faculty researchers on research methods, data analysis, and writing for publication; identify and prioritize any resources to support furthering research; the university will need to be strategic in identifying and allocating resources to fund research support; create new student research models and expand existing models- integrate research methods courses and small projects into undergraduate programs while expanding graduate research models. Models exist at various levels in international universities, which can be learned to support expansion at Fallujah.

The development of a robust, ongoing quality enhancement strategy will assist the university in fully utilizing data and maximizing results of the current quality assurance process; benchmark quality enhancement process to international accreditation requirements to systematically move the university closer to international accreditation requirements; and establish a unit or office of Institutional Research to gather and synthesize key data for senior administrators to make strategic decisions. This reflects the trends over time and provides a basis for informed data decisions. Consider the integration of data visualization software such as Tableau open-source software to synthesize key data and use it for decision making.

## Conclusion and recommendations: Next steps

The following insights on institutional change are important to keep in mind, as the UoF moves forward with plans for improvement:

1. Sustainable change will likely happen incrementally. To make this possible each step should have a limited, well-defined scope.
2. Public plans that lay out a process where the university community has an opportunity to participate in decision-making can be an effective tool to build commitment from various stakeholders.
3. Senior leaders need support. In a larger, complex university with significant levels of autonomy and accountability, senior leaders need professional staff and excellent data-collection capacity to make sustainable changes. The initial steps to further consider rebuilding include the following.
4. Conduct a full strategic planning process to revise and/or refine university goals and objectives, with a key focus on aligning college and departmental goals with overall institutional aims.
5. Benchmarking for institutional improvement at the institutional level for areas such as quality enhancement or at the departmental level, but mapping out concrete growth goals and steps toward improvement based on a U.S. university model.
6. Expand data collection and integration into institutional decision making across the entire institution.
7. Develop extensive institutional partnerships with the local and international community, including other governmental offices such as the Ministry of Planning or Ministry of Education; key private sector partnerships and engagement in areas such as Industry Advisory boards and research and development initiatives; and international and local partnerships with higher education institutions.
8. Expand footprint and engagement in the local community to meet critical need in Fallujah and the surrounding area.

## Ethical considerations

This study was reviewed and approved by the Research Ethics and Quality Assurance Committee at Fallujah University, Fallujah, Iraq. No approval reference number was issued because the work was conducted as part of an internal institutional capacity assessment mandated by the university’s leadership.

**Verbal informed consent was obtained from all participants**. Participants were selected because they occupied the top- and middle-level leadership roles responsible for institutional planning and quality improvement. Although participation formed part of their institutional responsibility in the post-war recovery and reassessment of the university, participation remained voluntary with no personal identifiers collected. The assessment involved minimal risk, and constituted an internal institutional effort to strengthen governance and academic resilience. All participants were informed of the study purpose and their right to decline or withdraw, and that their responses would remain confidential. **All procedures involving human participants were conducted in accordance with institutional ethical standards and the principles of the Declaration of Helsinki**.

## Data Availability

The dataset supporting this study was generated through an internal institutional assessment completed by dministrative staff at Fallujah University. Due to the confidential nature of the internal performance evaluations, the full dataset cannot be publicly released. Aggregated anonymized results are presented in the article. **For peer-review verification, the full dataset in its original Excel format can be provided confidentially to the journal’s editorial office upon request. For external researchers, non-sensitive aggregated summary data (e.g., domain-level HEICAT scores) are available upon reasonable request**. These summaries contain no identifiable information and comply with institutional confidentiality requirements. Requests may be directed to the corresponding author at: hamdan.obeed@uofallujah.edu.iq
