# Supplementary material for: Institutional Capacities Building of Universities in Post-Conflict Context: A Dynamic Managerial Capabilities Perspective: Early Post-Conflict Recovery Assessment (2019-2020)
Source: F1000Res. 2026 Apr 27;15:231. Originally published 2026 Feb 10. [Version 2] doi: 10.12688/f1000research.173628.2 (PMC13153773; doi:10.12688/f1000research.173628.2)
Supplement: Peer review history

# Reviewer report: Reviewer response for version 2

**DOI:** 10.5256/f1000research.198521.r479506

**Published:** 2026-05-07

**Authors:** Mohamed Ali Osman

**Affiliations:** 1 SIMAD University, Mogadishu, Somalia

**Comment 1: Strengthen the Theoretical–Empirical Linkage Using the DMC Framework Explicitly**

- **Issue:** The findings are presented as descriptive domain summaries (e.g., "Academic Operations were the strongest area") without systematically mapping results onto the three pillars of Dynamic Managerial Capabilities (DMC): human capital, social capital, and cognition. This weakens the theoretical contribution and makes the DMC framework feel more decorative than analytical.
- **Comment 2: Integrate Comparative Post-Conflict HE Literature & Bibliometric Trends**
- **Issue:** The literature review remains thin on comparative post-conflict higher education studies beyond the Somali and Libyan examples. It also misses recent bibliometric evidence showing that "sustainable development" and "institutional resilience" are growing themes in HEI research—directly relevant to post-conflict rebuilding.
- **Recommendation:** Add a short subsection or paragraph that:
  - Cites the requested bibliometric review (e.g., on HEIs and sustainable development, 2015–2023) to position your work within broader global trends of HEI capacity building.
  - Explicitly compare your Iraqi findings with the Somali case (Omar et al., 2025), noting shared challenges (e.g., curriculum fragmentation, weak QA, loss of intellectual capital) and unique contextual factors (e.g., degree of infrastructure destruction, nature of post-ISIS governance).
    Please do the following: papers 
  - 1. Assuring quality of teaching and learning in post-conflict higher education: The Case of Somalia.
  - 2.A bibliometric analysis of sustainable development research in higher education institutions (HEIs): key trends, global collaborations and influential contributions (2015–2023) 
- **Expected impact:** This positions your study not as an isolated case but as part of an emerging evidence base on post-conflict HE recovery, increasing its relevance and citability.
**Comment 3: Acknowledge and Mitigate the Single-Institution, Single-Timepoint Limitation**
- **Issue:** Although the authors now explicitly note that data are from 2019–2020 and frame the study as an “early post-conflict assessment,” the manuscript still implies broader applicability to “post-conflict Iraqi universities” (plural). No follow-up or longitudinal data are provided, and no cross-institutional comparison is attempted.
- **Comment 4: Enhance Feasibility of Recommendations for Severely Resource-Constrained Contexts**
- **Issue:** Several recommendations (e.g., “benchmark to a U.S. university,” “use Tableau software,” “implement electronic student management systems”) are disconnected from the post-conflict resource realities the study itself describes—such as destroyed labs, paper-based records, and no research office.

Is the work clearly and accurately presented and does it cite the current literature?

Partly

If applicable, is the statistical analysis and its interpretation appropriate?

Not applicable

Are all the source data underlying the results available to ensure full reproducibility?

Partly

Is the study design appropriate and is the work technically sound?

Partly

Are the conclusions drawn adequately supported by the results?

Partly

Are sufficient details of methods and analysis provided to allow replication by others?

No

Reviewer Expertise:

NA

I confirm that I have read this submission and believe that I have an appropriate level of expertise to confirm that it is of an acceptable scientific standard.

# Reviewer report: Reviewer response for version 1

**DOI:** 10.5256/f1000research.191459.r458537

**Published:** 2026-03-16

**Authors:** Ahmed Younis Alsabawy

**Affiliations:** 1 University of Mosul, Mosul, Nineveh Governorate, Iraq

Further statistical analysis is needed in this research, as the descriptive approach alone is insufficient for testing hypotheses and arriving at realistic conclusions. The discussion of the results should be based on a literature review. The researcher did not cite any sources in discussing the results, nor did they mention any previous studies in this field. The research problem should be more clearly defined. This can be achieved by including some quantitative indicators that demonstrate the existence of the problem. Simply citing international university rankings and the absence of Iraqi universities from them is insufficient. The introduction should generally address the research issue. However, we did not find any paragraph or statement specifically addressing this issue. The introduction should progress from general to specific in defining the research issue.

Is the work clearly and accurately presented and does it cite the current literature?

Yes

If applicable, is the statistical analysis and its interpretation appropriate?

Yes

Are all the source data underlying the results available to ensure full reproducibility?

Yes

Is the study design appropriate and is the work technically sound?

Partly

Are the conclusions drawn adequately supported by the results?

Partly

Are sufficient details of methods and analysis provided to allow replication by others?

Partly

Reviewer Expertise:

Business Administration

I confirm that I have read this submission and believe that I have an appropriate level of expertise to confirm that it is of an acceptable scientific standard.

# Reviewer report: Reviewer response for version 1

**DOI:** 10.5256/f1000research.191459.r461056

**Published:** 2026-03-13

**Authors:** Mohamed Ali Osman

**Affiliations:** 1 SIMAD University, Mogadishu, Somalia

Summary of the Article

 This study examines institutional capacity building at the University of Fallujah in Iraq following the defeat of ISIS (Da'esh). Drawing on Dynamic Managerial Capabilities (DMC) theory from strategic management literature—particularly the three pillars of managerial human capital (skills and knowledge), social capital (relationships and networks), and cognitive capital (belief systems and mental models)—the authors investigate how campus leaders contribute to rebuilding higher education in a post-conflict context.

 Using the Higher Education Institutional Capacity Assessment Tool (HEICAT), the research team collected data from 40 senior academic and administrative leaders at the University of Fallujah between December 2019 and January 2020. The assessment covered five domains: mission and strategic planning, academic operations, workforce development, research, and quality assurance. Data collection involved a 240-item questionnaire, semi-structured interviews, document analysis, and guided discussion sessions facilitated by ten domain specialists.

**Key Findings:**

- Institutional performance challenges stem less from material scarcity and more from underdeveloped managerial capabilities among campus leaders—particularly in strategic planning, research management, data-driven decision-making, and external relationship building
- While the university has basic systems in place across all domains, significant gaps exist in: strategic plan communication and alignment, research infrastructure and strategy, labor market engagement, data utilization for decision-making, and quality enhancement processes
- The research domain emerged as the weakest area, lacking institutional strategy, ethics policies, researcher support mechanisms, and research management infrastructure
- Academic operations was the strongest domain, with uniformly applied regulations and committed faculty despite conflict disruption
  Major Comments
  Strengths
**1. Timely and Important Research Question**
  The study addresses a genuine gap in the literature. Post-conflict higher education reconstruction is under-theorized and under-researched, yet critically important for long-term peacebuilding and development. The focus on leadership capabilities rather than just physical infrastructure offers a valuable corrective to reconstruction approaches that prioritize buildings over people. As Omar et al. (2025) note in their study of Somali higher education, "quality assurance in post-conflict contexts requires rebuilding not just systems but also the human capacity to operate them"—a finding this study empirically validates in the Iraqi context.
**2. Appropriate Theoretical Framework**
  Dynamic Managerial Capabilities theory is well-chosen for this investigation. The three dimensions (human, social, cognitive capital) provide a structured lens for analyzing leadership challenges without reducing complexity. The framework's emphasis on how managers sense, seize, and transform opportunities in changing environments maps well onto the post-conflict context where adaptability is essential.
**3. Rich Empirical Data**
  The HEICAT assessment generated detailed, domain-specific diagnostic information across five critical areas of university functioning. The combination of quantitative scoring with qualitative commentary provides both overview and depth. The inclusion of both "Strengths" and "Development Needs" for each domain offers a balanced picture that avoids either despair or triumphalism—important in post-conflict contexts where both acknowledging achievement and identifying gaps are essential.
**4. Insider Perspective**
  All authors are based at Iraqi universities (Al-Hamdaniya, Fallujah, Kirkuk), giving them embedded knowledge and access that external researchers would lack. This insider perspective enhances the authenticity of the findings and the appropriateness of the recommendations. The study demonstrates the value of locally-led research in post-conflict settings.
**5. Practical Recommendations**
  The discussion section offers 30+ concrete, actionable recommendations across the five domains. While some may require prioritization, the specificity is valuable for university leaders seeking guidance. The recommendations are grounded in the empirical findings rather than generic advice.
**6. Contribution to Emerging Literature**
  This study joins a small but growing body of work on post-conflict higher education. Recent scholarship on Somalia (Omar et al., 2025), has begun to build comparative knowledge about reconstruction challenges and strategies. This Iraqi case adds an important data point, particularly given the severity of ISIS destruction and the unique challenges of the Iraqi higher education context.
  Weaknesses and Areas for Improvement
**1. Data Timing and Contextualization (Must Address)**
  The data were collected from December 2019 to January 2020—over six years before publication. This gap is not acknowledged or justified in the current manuscript. Post-conflict contexts are highly dynamic; institutional leadership, resources, and challenges may have changed substantially since 2020. The University of Fallujah may have progressed, stagnated, or faced new challenges in the intervening years.
**Recommendation:** Either (a) reframe the manuscript as "Early Post-Conflict Recovery Assessment (2019-2020)" with explicit acknowledgment that findings represent that specific historical moment, or (b) add a postscript or follow-up section describing developments since 2020 (if authors remain connected to the university and can provide updated information). At minimum, the limitations section must address this timing issue transparently.
**2. Methodological Transparency (Must Address)**
  Critical methodological details are missing, preventing replication and full evaluation:
- Sampling: How many approached vs. participated? Response rate? Gender composition? (Likely male-dominated—this should be acknowledged)
- HEICAT implementation: The 240-item questionnaire was not fully administered to all participants, with facilitators "tailoring" questions. How were items selected? How can data be compared across participants with inconsistent instrument administration?
- Facilitators: How were the 10 domain facilitators selected and trained? What were their qualifications?
- Analysis: How were qualitative data coded? How were multiple data sources synthesized into scores? Was there inter-rater reliability checking?
- **Recommendation:** Provide a methodological appendix with complete protocols, sampling details, facilitator qualifications, and analysis procedures. If the full HEICAT instrument is proprietary, include a detailed summary of domains and sample items.
**3. Theoretical-Empirical Disconnect (Must Address)**
  The findings are presented as descriptive domain summaries without explicit connection to the DMC framework. The reader must infer which findings relate to human vs. social vs. cognitive capital. This weakens the theoretical contribution and makes the DMC framework feel decorative rather than analytical.
**Recommendation:** Restructure the findings section around the three DMC dimensions. For each key finding, explicitly state: "This reflects underdeveloped managerial human capital because..." or "This indicates cognitive limitations among leaders as evidenced by..." Add a conceptual framework diagram showing hypothesized relationships between DMC dimensions and institutional outcomes.
**4. Ethics Documentation (Must Address)**
  The ethics statement is concerning: "No approval reference number was issued because the work was conducted as part of an internal institutional capacity assessment mandated by the university's leadership." For indexing purposes, research involving human subjects requires formal ethics review regardless of institutional mandate. "Verbal informed consent" is weaker than written consent and may not meet journal standards. Participants were senior leaders—was there implicit pressure to participate?
**Recommendation:** Either seek retroactive ethics approval from an appropriate body or add detailed description of consent procedures, confidentiality protections, and steps taken to ensure voluntary participation. Explicitly acknowledge limitations of the current ethics documentation.
**5. Literature Gaps (Should Address)**
  While foundational DMC literature is well-covered, the manuscript misses:
- Recent DMC scholarship (2016-2025) that could inform the analysis
- Post-conflict higher education literature beyond the minimal three sources cited
- Iraqi higher education context (history, sanctions impact, post-2003 developments)
- Comparative studies of other post-conflict university reconstructions
- **Recommendation:** Add 10-15 sources on post-conflict higher education, including recent work like Omar et al. (2025) on Somalia. Update DMC citations to include scholarship from the last decade. Add background on Iraqi higher education to contextualize the case.
**6. Recommendation Feasibility (Should Address)**
  Some recommendations (e.g., "benchmark to a U.S. university," "Tableau software," "electronic student management systems") seem disconnected from the post-conflict resource constraints the study itself emphasizes. How feasible are these in a context where basic infrastructure may still be lacking?
**Recommendation:** Prioritize recommendations (high/medium/low) with implementation considerations. Acknowledge resource constraints explicitly and suggest low-cost alternatives where possible (e.g., open-source software options, regional rather than U.S. benchmarking).
**7. Missing Stakeholder Voices (Should Address)**
  The study includes only senior leaders. Junior faculty, students, community members, and private sector representatives, all relevant to capacity assessment, are excluded. This limits the diagnostic's comprehensiveness.
**Recommendation:** Acknowledge this limitation explicitly and note that future research should include multiple stakeholder perspectives.
  The authors would strengthen their literature review by engaging with recent scholarship on post-conflict higher education quality assurance. I recommend citing:
  [Refer to reference 1]

Is the work clearly and accurately presented and does it cite the current literature?

Partly

If applicable, is the statistical analysis and its interpretation appropriate?

Not applicable

Are all the source data underlying the results available to ensure full reproducibility?

Partly

Is the study design appropriate and is the work technically sound?

Partly

Are the conclusions drawn adequately supported by the results?

Partly

Are sufficient details of methods and analysis provided to allow replication by others?

No

Reviewer Expertise:

NA

I confirm that I have read this submission and believe that I have an appropriate level of expertise to confirm that it is of an acceptable scientific standard, however I have significant reservations, as outlined above.

## References

[1] : Assuring quality of teaching and learning in post-conflict higher education: The Case of Somalia. *Cogent Education* .2025;12(1) : 10.1080/2331186X.2025.2543039 10.1080/2331186X.2025.2543039

# Response

**Published:** 2026-04-06

**Authors:** Hamdan O. Mansoor

**Affiliations:** Business Administration, University of Fallujah, Al-Fallujah, Al Anbar Governorate, Iraq

**Dear Dr. Mohamed Ali Osman, **SIMAD University, Mogadishu, Somalia,

 On behalf of the authors, I would like to sincerely thank you for your thorough, insightful, and constructive review. Your comments have been highly valuable in improving the clarity, methodological rigor, and theoretical contribution of this study. We have carefully revised the manuscript to address all the points raised, and our detailed responses are provided below.

 1. **Data Timing and Contextualization (Must Address)**

**Comment: **The data (2019–2020) are outdated and not contextualized.

**Response:**

 In response, we have reframed the manuscript as an early post-conflict institutional capacity assessment (2019–2020). This change is now reflected in the title.

 2. **Methodological Transparency**

**Comment:** Key methodological details are missing (sampling, HEICAT implementation, facilitators, analysis, etc.).

**Response:**

 we have developed a comprehensive Methodological Appendix (Supplementary Materials), which includes:

- HEICAT domains and illustrative items
- Detailed data collection protocols
- Facilitator roles and qualifications
- Scoring framework and analytical procedures
- Supporting evidence (including SWOT-based analysis)

3. **Ethics Documentation**

**Comment:** Ethical procedures are unclear; concerns about consent and lack of formal approval.

**Response:** The ethics section has been substantially revised to improve clarity and transparency.

 Further, The paper now clearly stated as limitations in relation to international publication standards.

 4. **Literature Gaps**

**Comment:**

 Limited engagement with recent literature and post-conflict higher education research.

**Response: **We Added literature on post-conflict higher education systems, including comparative cases, Integrated reviewer-recommended studies (e.g., Somalia case, Libya context, and post-conflict reconstruction literature). Additionally, the literature review has been expanded to incorporate recent scholarship on post-conflict higher education and dynamic capabilities, including the study by Omar, Osman, and Abdi (2025) on quality assurance in Somali higher education, which provides a comparative post-conflict perspective.

 Thank you very much for your insightful and constructive feedback.

 Corresponding Author
